# “Case report: Integrating aerobic activity in post-surgical management of plurifragmentary distal clavicle fractures - A holistic approach to pain modulation and recovery”

**DOI:** 10.1016/j.ijscr.2023.109024

**Published:** 2023-11-10

**Authors:** Roberto Tedeschi

**Affiliations:** Department of Biomedical and Neuromotor Sciences, Alma Mater Studiorum, University of Bologna, Bologna, Italy

**Keywords:** Clavicle fractures, Surgical intervention, Aerobic activity, Orthopedic care, Case report

## Abstract

**Introduction:**

Clavicle fractures, particularly at the distal end, are common orthopedic injuries. While surgical interventions are often prioritized, the role of aerobic activity in post-operative pain modulation remains underexplored.

**Case presentation:**

A 34-year-old male presented with a plurifragmentary distal clavicle fracture following a direct trauma during a soccer match. Initial pain was severe (NPRS 6/7), with restricted shoulder mobility. Radiographic examinations confirmed the fracture. Following orthopedic consultation, the patient underwent surgical fixation using plate and screws. Post-operatively, pain management was uniquely addressed using low-impact aerobic activities, progressing from walking to running. At the 7-year follow-up, the patient reported optimal functional outcomes with a Disabilities of the Arm, Shoulder, and Hand (DASH) score of 0, highlighting the success of the combined approach.

**Clinical discussion:**

The surgical intervention ensured anatomical alignment and stability, crucial for the healing of plurifragmentary fractures. The innovative approach of using aerobic activity for pain modulation post-surgery showcased significant pain reduction without consistent reliance on medications.

**Conclusions:**

This case emphasizes the potential benefits of a comprehensive approach to clavicle fracture management. By integrating surgical intervention with aerobic activity for pain modulation, patients can achieve optimal long-term recovery and improved quality of life.

## Highlights

• Distal clavicle fracture managed with surgical fixation.
• Aerobic activity effectively modulated post-op pain.
• Case emphasizes holistic orthopedic treatment approach.

## 1 Introduction

Clavicle fractures are prevalent injuries encountered in the Emergency Department (ED). Predominantly seen in children and young adults, they constitute about 4 % of all fractures presented in the ED and make up 44 % of shoulder girdle fractures [1,2]. The primary cause, accounting for 87 %, is a fall onto the shoulder. Other causes include direct trauma to the clavicle or a fall onto an outstretched hand (FOOSH) [3]. Atraumatic clavicle fractures are uncommon, with a Swedish study reporting a mere 0.7 % occurrence due to non-traumatic reasons, primarily pathologic fractures [4]. The middle third of the clavicle is the most commonly fractured area, representing 70–80 % of such injuries [5]. The distal (lateral) third follows, accounting for 17–25 % [6], while the proximal third fractures are rare, constituting less than 5 % [6].

In most cases, nondisplaced clavicle fractures can be treated conservatively, resulting in satisfactory outcomes and full functional recovery [7,8]. Surgical intervention, for specific patients, has demonstrated enhanced functional results and a decreased nonunion rate [[9], [10], [11]]. Un obiettivo primario nella gestione delle fratture della clavicola è ridurre al minimo il rischio di pseudoartrosi e di malunione sintomatica, poiché queste complicanze possono influenzare significativamente gli esiti a lungo termine del paziente [12].

In this case report, we aim to emphasize the significance of low-frequency aerobic activity in pain modulation for clavicle fractures. While the majority of the literature focuses on the anatomical and surgical aspects of these fractures, the role of aerobic exercises in pain management remains largely unexplored [13]. We believe that addressing pain is not solely about pharmacological interventions; physical activity plays a crucial role in holistic patient care. Furthermore, this report provides a unique long-term perspective. We offer a 7-year follow-up on the patient, evaluating both functional outcomes and pain levels. Such long-term insights are rare in the literature, especially when combined with an alternative pain management approach.

## 2 Case presentations

**Fig. 1:**
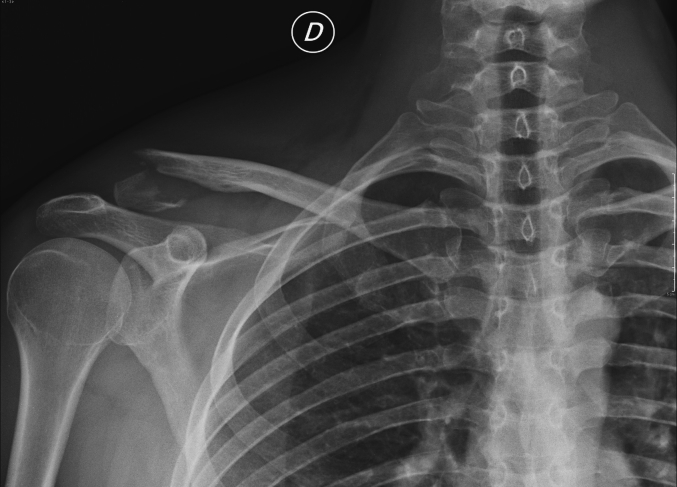
Xray clavice.

**Fig. 2:**
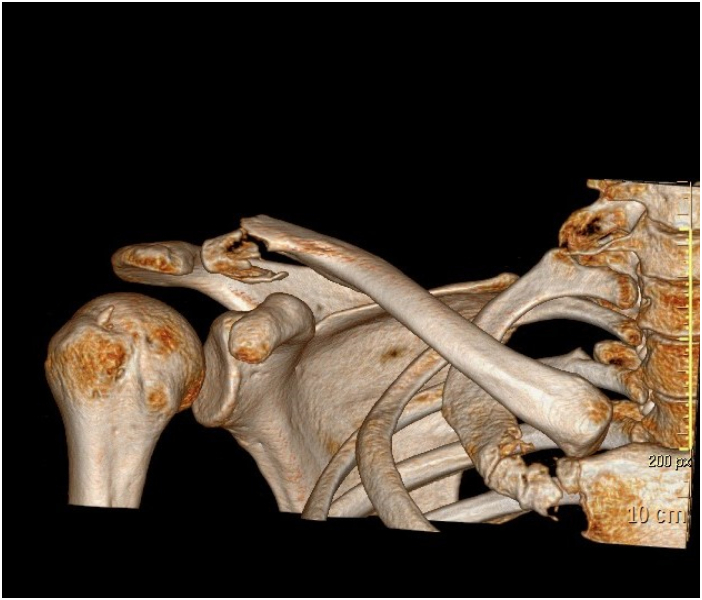
TC clavice.

**Fig. 3:**
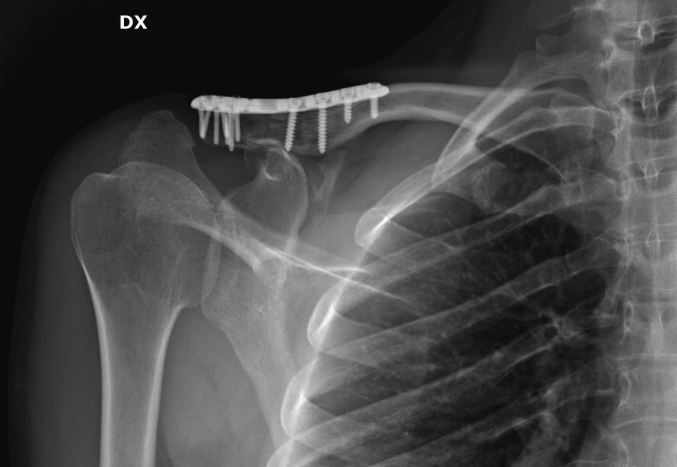
Xray clavice after surgery.

A 34-year-old European Caucasian male patient presented to the emergency department with pain and immobility in his right shoulder following a direct trauma the previous evening while playing a soccer match. The patient had no history of previous fractures or concurrent pathologies. However, he had a minor alteration in von Willebrand factor, consistent with his blood type O positive. The patient is alert and cooperative, reporting no other pain. Upon physical examination, there is severe tenderness (Numeric Pain Rating Scale [14] - NPRS 6/7) with already visible bruising on the right shoulder. Both active and passive shoulder mobility are impossible to perform. Radiographic examinations (X-ray) (Fig. 1) are requested, which confirm a multifragmentary fracture of the distal margin of the right clavicle. After consultation with an orthopedic specialist and considering the patient's profession as a physiotherapist, it was decided to surgically fix the fracture using a plate and screws. A CT(Fig. 2) scan was conducted to better assess the fracture. The scan revealed a multifragmentary fracture of the lateral end of the clavicle, lateral to the coracoid and trapezoid ligaments, with partial displacement. Upon discharge, the patient was provided with a brace and a sling, to be removed only for dressing changes, hygiene care, and physiotherapy. He was advised to mobilize the elbow twice daily and to take pain relief medication if needed. One month post-surgery, a radiographic examination (Fig. 3) showed initial signs of bone consolidation. The shoulder was mobile on the scapular plane. Pain was managed with low-impact aerobic activity, specifically walking for 30–60 min daily without the need for medication (NPRS: 1). Active range of motion (ROM) was measured as follows: flexion approximately 90°, abduction 60°, internal rotation 20°, and external rotation 35°. The patient was evaluated using the DASH [15] score, which was 70/100. At the second follow-up, two months post-surgery, pain was managed with aerobic activity, specifically running for 30–45 min daily without the need for medication (NPRS: 0). Active range of motion (ROM) was as follows: flexion 140°, abduction 100°, external rotation 45°, and internal rotation 30°. Rotator cuff tests (Jobe, External Rotation Lag Sign, Lift-off Test, Internal Rotation Lag Sign, Yocum) were all negative. Strength was evaluated using the Medical Research Council (MRC) scale, with the biceps brachii scoring 4/5 and the triceps brachii scoring 4/5. The patient was evaluated using the DASH score, which was 40/100. Seven years later, the plate remains in place and causes some discomforts. These include tenderness when lying on the right side, hypoesthesia around the scar, and a stinging sensation when lifting weights over 5 kg with an extended arm in anterior flexion repeatedly. The patient also occasionally experiences discomfort from vibrations, such as when cycling on uneven terrain. ROM full active and passive. Rotator cuff tests (Jobe [16], External Rotation Lag Sign [17], Lift-off Test [18], Internal Rotation Lag Sign [19], Yocum [20]) were all negative. Strength was evaluated using the MRC (Medical Research Council) scale, with the biceps brachii scoring 5/5 and the triceps brachii scoring 5/5. The patient was evaluated using the DASH score, which was 0/100. This case study adheres to the SCARE [21] (Surgical Case Report) guidelines for reporting surgical case studies. The SCARE guidelines aim to enhance the transparency and completeness of reporting surgical cases, providing a structured framework that facilitates accurate communication and assessment of surgical experiences.

## 3 Clinical findings

○ Subjective Reports:

Seven years post-surgery, the patient provided the following subjective feedback regarding his condition:

1. Plate Discomfort: The plate, which was not removed, occasionally causes discomfort. This is particularly noticeable when the patient lies on his right side.
2. Sensory Changes: There is a noticeable hypoesthesia (reduced sensitivity) around the scar area.
3. Activity-Related Discomfort: A distinct stinging sensation is felt when the patient lifts weights exceeding 5 kg with a fully extended arm in anterior flexion, especially if the action is repeated multiple times.
4. Vibration Sensitivity: Activities that involve vibrations, such as cycling on rough terrains, sometimes cause discomfort.
5. Strength and Mobility: Despite the aforementioned discomforts, the patient's strength remains relatively good, with the biceps brachii scoring 4/5 and the triceps brachii 5/5 on the MRC scale. The rotator cuff tests were all negative, indicating no significant impairment in the shoulder's rotator cuff muscles.
6. Daily Activities: The patient mentioned that while most daily activities can be performed without significant pain, certain actions, especially those involving lifting or vibrations, can trigger the discomfort.

○ Physical Examination:
○ Inspection:
  • Scar: A well-healed surgical scar was visible over the right clavicle area.
  • Posture: The patient's shoulder alignment appeared normal without any visible deformities or asymmetry.
  • Skin Changes: There was no sign of inflammation, redness, or swelling around the surgical site.
○ Palpation:
  • Tenderness: The patient reported tenderness when pressure was applied over the plate area, especially when lying on the right side.
  • Temperature: The skin temperature around the surgical site was normal, indicating no active inflammation.
  • Consistency: No unusual masses or lumps were felt around the surgical site.
○ Range of Motion (ROM):
  • Active ROM: Flexion was measured at 180°, abduction at 180°, external rotation at 85°, and internal rotation at 90°.
  • Passive ROM: Similar to active ROM, with no significant restrictions noted.
○ Strength Testing:
  • Biceps Brachii: Scored 5/5 on the MRC scale.
  • Triceps Brachii: Scored 5/5 on the MRC scale.
○ Special Tests:
  • Rotator Cuff Tests: All tests, including Jobe, External Rotation Lag Sign, Lift-off Test, Internal Rotation Lag Sign, and Yocum, were negative, indicating no significant impairment in the shoulder's rotator cuff muscles.
○ Neurovascular Examination:
  • Sensory: Hypoesthesia was noted around the scar area.
  • Motor: No deficits were observed.
  • Vascular: Pulses were palpable and symmetrical, with no signs of vascular compromise.
○ Instrumental Investigations:
○ Radiographic Examination (X-ray):
  • Findings: Initial post-operative X-rays showed a well-aligned fracture with the plate and screws in the correct position. At the seven-year mark, radiographs displayed signs of complete bone consolidation without any signs of plate migration or screw loosening.
  • Bone Density: No signs of osteopenia or osteoporosis were observed around the fracture site.

Computed Tomography (CT) Scan:

Findings: Prior to the surgical intervention, a detailed CT scan was conducted to better understand the fracture's intricacies and determine the most appropriate surgical approach. The scan revealed a multifragmentary fracture of the lateral end of the clavicle, lateral to the coracoid and trapezoid ligaments, with partial displacement.

The Disabilities of the Arm, Shoulder, and Hand (DASH) score is a self-reported questionnaire used to measure physical function and symptoms in individuals with musculoskeletal disorders of the upper limb. The DASH score provides clinicians and researchers with a standardized measure to assess the patient's health status and change in health over time.

Components:

1. Questionnaire: The DASH questionnaire consists of 30 items. Each item asks the patient how much difficulty they had in performing different physical activities in the past week due to their arm, shoulder, or hand problem. Activities include daily tasks such as dressing, cooking, and sleeping.
2. Scoring: Each question is scored on a scale of 1 to 5. A score of 1 indicates “no difficulty,” while a score of 5 indicates “unable to do.” The scores are then summed and averaged, producing a score out of 100.
3. Interpretation: A higher score indicates a greater disability. A score of 0 would indicate no disability, while a score of 100 would indicate the most severe disability.

In the presented case, the patient's DASH score of 70/100 indicates a significant level of functional impairment, suggesting that the patient's upper limb disorder has a considerable impact on their daily activities and quality of life.

Therapeutic intervention

Phases of Intervention:

1. Initial Phase - Walking:
  ○ Duration: 30–60 min daily.
  ○ Intensity: A steady pace of 6.5 km/h.
  ○ Objective: Introduce the patient to a consistent aerobic routine, promoting blood circulation, enhancing endorphin release, and gradually improving cardiovascular fitness without stressing the injured area.
2. Advanced Phase - Running:
  ○ Duration: 30–45 min daily.
  ○ Intensity: A faster pace of 12 km/h.
  ○ Objective: As pain levels decreased and functional capacity improved, the intensity of aerobic activity was increased to further promote cardiovascular health and endorphin release.
3. Orthopedic and Physiotherapy Interventions:
  ○ Strength Training: Based on orthopedic recommendations, specific exercises were introduced to improve muscle strength around the shoulder and upper limb.
  ○ Mobility Exercises: Standard physiotherapy protocols were followed to enhance the range of motion and flexibility of the shoulder joint.
  ○ Pain Management: While strength and mobility exercises were crucial, special attention was given to pain modulation. The combination of aerobic activity with physiotherapy interventions ensured a holistic approach to pain management.

Benefits of the Comprehensive Intervention:

• Pain Reduction: The combination of aerobic exercises and physiotherapy interventions effectively reduced pain levels by stimulating the release of endorphins and improving joint mobility.
• Improved Cardiovascular Health: Regular aerobic activity, from walking to running, strengthened the heart and lungs.
• Enhanced Muscle Strength and Mobility: With the guidance of orthopedic and physiotherapy recommendations, the patient achieved optimal muscle strength and joint mobility.
• Mental Well-being: Aerobic exercises, combined with the sense of achievement from regaining strength and mobility, contributed to improved mental health.
• Reduced Reliance on Medications: Effective pain modulation through the combined approach reduced the need for pain medications, minimizing potential side effects.

FOLLOW-UP AND OUTCOMES

1. Short-Term Follow-Up (1–3 months post-surgery):
  • Pain Assessment: The patient reported a significant reduction in pain levels, with an NPRS score dropping from 6/7 initially to 1 post-intervention. The pain modulation achieved through aerobic activity played a pivotal role in this improvement.
  • Functional Assessment: The patient's range of motion showed marked improvement, with active flexion reaching 90°, abduction at 60°, internal rotation at 20°, and external rotation at 35°.
  • DASH Score: The score was recorded at 70/100, indicating some level of functional impairment but showing potential for further improvement.
2. 2. Mid-Term Follow-Up (2–6 months post-surgery):
  • Pain Assessment: The patient reported no pain (NPRS: 0) during daily activities, attributing the pain relief to the combination of aerobic exercises and physiotherapy interventions.
  • Functional Assessment: There was a further increase in the range of motion with flexion at 140°, abduction at 100°, external rotation at 45°, and internal rotation at 30°. Strength assessments using the MRC scale showed improvements in the biceps brachii (4/5) and the triceps brachii (4/5).
  • Activity Level: The patient progressed from walking to running for 30–45 min daily at a pace of 12 km/h, further emphasizing the role of aerobic activity in pain modulation and overall recovery.
3. Long-Term Follow-Up (7 years post-surgery):
  • Pain Assessment: The patient reported occasional discomfort when sleeping on the right side and a tingling sensation during specific activities, such as lifting weights in anterior flexion. However, these episodes were infrequent and manageable.
  • Functional Assessment: The patient maintained a good range of motion in the shoulder, with strength assessments showing sustained improvements. The rotator cuff tests, including Job, External Rotation Lag Sign, Lift-off Test, Internal Rotation Lag Sign, and Yokum, were all negative, indicating no significant issues with the rotator cuff muscles.
  • Instrumental Assessment: Radiographic examinations revealed that the plate remained in place, with no signs of migration or loosening. The bone showed complete consolidation, and there were no signs of osteoporosis or osteopenia.
  • DASH Score: At the 7-year mark, the patient's DASH score was recorded as 0, indicating no disability and reflecting optimal functional outcomes and a high quality of life.
  • In summary, the patient's follow-up sessions highlighted the success of the comprehensive therapeutic intervention. The combination of aerobic activity, orthopedic guidelines, and standard physiotherapy not only improved the patient's physical function but also effectively managed pain. The DASH score of 0 at the 7-year follow-up is a testament to the patient's complete recovery and return to a normal, active lifestyle.

## 4 Discussion

The management of clavicle fractures, especially those that are plurifragmentary and located at the distal end, presents a clinical challenge. The presented case underscores the significance of a comprehensive approach, combining surgical intervention with post-operative pain modulation through aerobic activity, and its long-term benefits [22]. The decision to opt for surgical intervention in the form of plate and screw fixation was pivotal. Given the nature of the fracture - plurifragmentary and at the distal end - conservative management might not have yielded optimal outcomes. The surgical approach ensured anatomical alignment, providing a stable foundation for the healing process. This is particularly crucial for fractures lateral to the coracoclavicular ligaments, where non-operative management can lead to higher rates of non-union or malunion [5]. The surgical intervention, in this case, laid the groundwork for successful long-term recovery. Post-operative pain management is often dominated by pharmacological interventions. However, this case highlights the potential of aerobic activity as an effective tool for pain modulation [22,23]. Starting with low-impact activities like walking and gradually progressing to running, the patient experienced significant pain relief without the consistent need for pain medications. Aerobic exercises are known to release endorphins, the body's natural painkillers, and enhance blood circulation, promoting healing. This non-pharmacological approach not only reduced potential side effects from medications but also contributed to the patient's overall well-being and cardiovascular health [23]. The long-term follow-up, especially the DASH score of 0 at the 7-year mark, is a testament to the success of the combined approach. It's noteworthy that while the patient achieved optimal functional outcomes, occasional discomfort was reported, underscoring the importance of continuous monitoring and potential interventions even years after the initial injury. The case offers valuable insights for clinicians dealing with similar fractures. While surgical intervention remains the cornerstone for certain types of clavicle fractures, the post-operative phase's management can significantly influence long-term outcomes. Incorporating aerobic activities into the rehabilitation protocol can offer dual benefits - pain modulation and improved cardiovascular health. Moreover, this case emphasizes the importance of long-term follow-ups, ensuring that any residual or emerging issues are promptly addressed. In conclusion, the presented case offers a holistic view of managing complex clavicle fractures. The combination of surgical precision, innovative pain management through aerobic activity, and diligent follow-ups ensured optimal recovery, emphasizing the need for a comprehensive approach in orthopedic care.

## Ethical approval

Ethical approval is not a requirement at our institution for reporting individual cases or case series.

## Funding

Authors state no funding involved.

## Author contribution

RT contributed to conception and design of the study; RT to data acquisition, RT to data analysis and interpretation; RT contributed to draft the manuscript; RT contributed to the critical revision for important intellectual content. All authors read and approved the final version of the manuscript.

## Guarantor

Roberto Tedeschi

## Consent

Written informed consent was obtained from the patient for publication of this case report and accompanying images. A copy of the written consent is available for review by the Editor-in-Chief of this journal on request.

## Conflict of interest statement

Authors state no conflict of interest.
